# A Gesture-Controlled Rehabilitation Robot to Improve Engagement and Quantify Movement Performance

**DOI:** 10.3390/s20154269

**Published:** 2020-07-31

**Authors:** Ava D. Segal, Mark C. Lesak, Anne K. Silverman, Andrew J. Petruska

**Affiliations:** 1 M3Robotics and Functional Biomechanics Laboratories, Department of Mechanical Engineering Colorado School of Mines, Golden, CO 80401, USA; asilverm@mines.edu (A.K.S.); apetruska@mines.edu (A.J.P.); 2 Army Cyber Institute, West Point, NY 10996, USA; mark.lesak@westpoint.edu

**Keywords:** gesture control, movement performance, feedback, telerehabilitation, game therapy, motor learning, wearable sensors

## Abstract

Rehabilitation requires repetitive and coordinated movements for effective treatment, which are contingent on patient compliance and motivation. However, the monotony, intensity, and expense of most therapy routines do not promote engagement. Gesture-controlled rehabilitation has the potential to quantify performance and provide engaging, cost-effective treatment, leading to better compliance and mobility. We present the design and testing of a gesture-controlled rehabilitation robot (GC-Rebot) to assess its potential for monitoring user performance and providing entertainment while conducting physical therapy. Healthy participants (*n* = 11) completed a maze with GC-Rebot for six trials. User performance was evaluated through quantitative metrics of movement quality and quantity, and participants rated the system usability with a validated survey. For participants with self-reported video-game experience (*n* = 10), wrist active range of motion across trials (mean ± standard deviation) was 41.6 ± 13° and 76.8 ± 16° for pitch and roll, respectively. In the course of conducting a single trial with a time duration of 68.3 ± 19 s, these participants performed 27 ± 8 full wrist motion repetitions (i.e., flexion/extension), with a dose-rate of 24.2 ± 5 ^reps^/_min_. These participants also rated system usability as excellent (score: 86.3 ± 12). Gesture-controlled therapy using the GC-Rebot demonstrated the potential to be an evidence-based rehabilitation tool based on excellent user ratings and the ability to monitor at-home compliance and performance.

## 1. Introduction

Physical therapy, a non-invasive treatment to address mobility impairments, requires high doses of repetitive exercise and coordinated movements to elicit improved function [1]. The monotony of these repetitive tasks contributes to low motivation and inconsistent participation, especially for home-based programs, with adherence to therapy ranging between 30 and 60% [2,3]. Furthermore, standard therapy sessions often fall short of the hundreds of repetitions required for neuroplastic motor recovery [4]. Without adequate training, functional deficits persist that hinder recovery. For example, impairments often linger in people with acquired brain injury due to a gradual recovery process requiring prolonged treatment to effectively target upper limb motor control deficits [5]. Long-term deficits also persist in people post-stroke, with 67% reporting continued disuse after four years [6]. As motivation and engagement are critical for successful rehabilitation [7] and compliance to post-acute rehabilitation care is associated with improved functional outcomes [8], engaging at-home therapy interventions are warranted for prolonged and effective treatment [9]. Rehabilitation through technology-based game therapy has the potential to improve compliance and dosage [10], quantify human motor performance [11], target motor control deficits [5], and improve engagement in various populations [12]. This type of therapy has also shown potential for motor learning, with a portion of this work previously presented at the IEEE Engineering in Medicine and Biology Conference [13].

Technology-based rehabilitation may improve standard therapy outcomes with motivating tasks and user feedback, which invite the repetition of intensive motion and motor learning. For example, a sensor-based therapy system improved arm-hand function in stroke survivors after the motor recovery plateau phase, which typically occurs six months post-therapy [14]. Specifically, they showed progressive and challenging task-oriented arm training with quantitative feedback improved upper-limb performance post-stroke by more than 10%. Furthermore, robot-assisted gait training elicited improved function in children with neurological gait disorders using virtual reality to promote engagement [15]. Video-game therapy also improved muscle activation for upper limb prosthesis control compared to the baseline, and users reported racing games as more engaging compared to rhythm games [16]. Furthermore, virtual reality-based rehabilitation supports retraining of movement planning and motor control, which could promote recovery from acquired brain injury [5]. Although the underlying mechanisms for the achieved recovery remain somewhat elusive, evidence suggests rehabilitation techniques that incorporate brain and body engagement promote neural plasticity [17], which is driven by user-initiated neuromuscular control.

Gesture control provides intuitive and efficient solutions in robotics, with specific applications including unmanned ground vehicles for military surveillance [18] and improved manufacturing safety and efficiency [19], robotic arm manipulation for prosthetic devices [20], robot-assisted surgeries [21], and robotic nursing-care assistants [22]. Rehabilitation robots that facilitate therapy with quantitative measures of movement performance have demonstrated potential as complementary assessment tools; however, they are typically limited to a clinical setting and are not easily translated to the home environment [23]. Despite the promising advantages related to gesture-based therapies, high complexity and cost [24], as well as a lack of portability [25] remain barriers to use. In addition, excessive screen time may be detrimental to health [26], with potentially adverse physical, psychological, and neurological effects [27]. Therefore, alternative game therapies that are engaging and that do not require screen use are needed.

**Figure 1:**
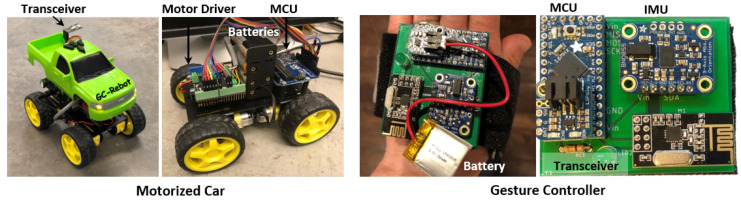
The gesture-controlled rehabilitation robot (GC-Rebot) includes a (left) motorized car with a microcontroller (MCU), motor driver, and radio transceiver, which are powered by eight AA batteries, and a (right) gesture controller with an IMU, radio transceiver, and miniature MCU, which are powered by a rechargeable lithium ion battery.

The goal of this work is to assess the feasibility of a portable, low-cost, easy-to-use, and engaging alternative to standard physical therapy for evidence-based rehabilitation. We designed a gesture-controlled rehabilitation robot (GC-Rebot) with wireless body movement control (Figure 1) to mimic standard active range of motion exercises while also promoting brain and body engagement through hand-eye coordination training without the use of a screen. The following sections provide the methodological details of the GC-Rebot system design, human subject experiments, and data analysis. Motor learning based on quantitative metrics of movement quality and quantity and potential engagement through system usability and perceived effort are then assessed for a healthy population to characterize the feasibility of this approach. In addition, as learning can be affected by the skill level of the performer [28], we sought to compare users of varying spatial mapping skill levels based on prior video-game experience during a standardized training session with the GC-Rebot.

## 2. Methods

### 2.1. System Design

**Figure 2:**
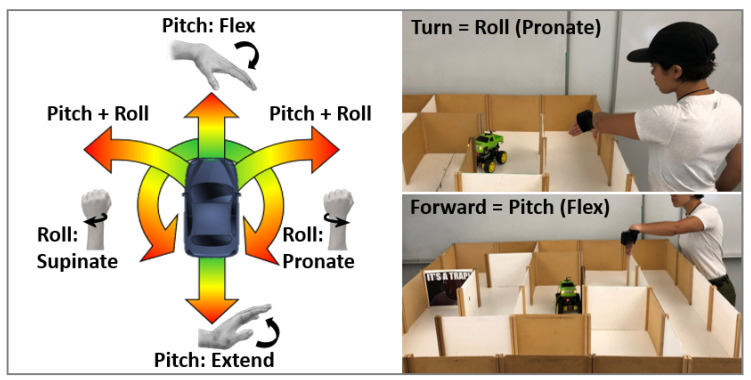
Proportional car control for left-handed operations. (Left) Mapping between user gestures and car movements. Wrist flexion-extension (pitch) produces forward/backward motion; pronation-supination (roll) produces on-the-spot turning; and flexion combined with roll produces gradual arching turns. (Right) Typical hand gestures for translational and rotational car control during a testing session.

The GC-Rebot system consists of a hand-mounted gesture controller and a motorized car (Table 1, Figure 1). The gesture controller (55 × 45 mm, mass = 26 g) includes an inertial measurement unit (IMU), which senses 3D linear accelerations and angular velocities, processes the signals in real-time with an embedded system, and transmits them wirelessly at 10 Hz. A commercially available four-wheel-drive platform (13.5 × 20 cm) was retrofitted with an Arduino microcontroller that provides proportional motor control of the wheel velocity based on the user’s gestures, as depicted in Figure 2.

An additional wireless IMU (MTw Awinda, Xsens Technologies B.V., Netherlands; 47 × 30 × 13 mm; mass = 16 g), powered with an integrated LiPo battery and sampled via Bluetooth at 100 Hz (Xsens MT Manager 4.6, Windows 10) according to the manufacturer guidelines, provides accurate 3D orientation using on-board sensor fusion to compensate for sensor drift [29]. Mounted in parallel with the GC-Rebot gesture controller, this secondary IMU provides data to assess the user’s motor performance.

The controlling software is split between the two platforms. The gesture-controller program uses the manufacturer provided data-fusion algorithms to extract the pitch and roll angles and then transmits them wirelessly to the car at 10 Hz. The car’s program receives the transmitted data, also at 10 Hz, and maps hand orientations (pitch: $\theta_{P}$, roll: $\theta_{R}$) to the car’s linear (*v*) and angular (ω) velocities according to,

(1) $$v=\mathrm{sat}\left(\theta_{P},-90^{{}^{\circ}},90^{{}^{\circ}}\right)*\frac{v_{max}}{90^{{}^{\circ}}}$$

(2) $$\omega =\mathrm{sat}\left(\theta_{R},-90^{{}^{\circ}},90^{{}^{\circ}}\right)*\frac{\omega_{max}}{90^{\circ }}$$

where sat(·) is the saturation function, the maximum desired car linear velocity ($v_{max}$) is 0.3 ± 10% m/s, and the maximum desired angular velocity ($\omega_{max}$) is 1.8 ± 10% rad/s. These velocities were chosen through pilot testing to provide a reasonable balance between precision and speed for the given task.

**Figure 3:**
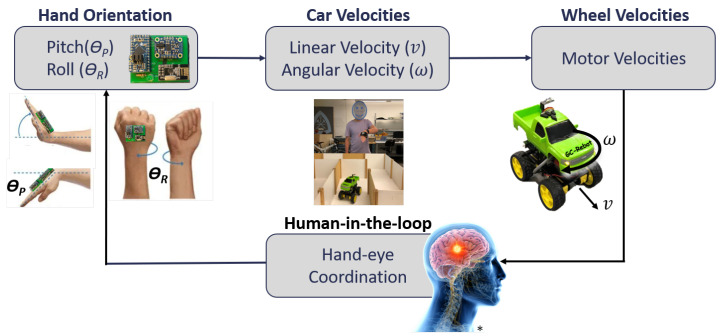
GC-Rebot system design schematic. Hand orientations are mapped to car velocities (v,ω) and converted to motor velocities for proportional open-loop motor control. Hand-eye coordinated movements close the control loop, providing feedback to the system through changes in wrist pitch and roll (* brain image courtesy of [31]).

After mapping gesture motions to motor velocities, directional control is established with conditional statements based on the sign and ratio of pitch and roll (Figure 2). Subsequently, user hand-eye coordination provides feedback to the system through human-in-the-loop control, which incorporates perception, cognition, planning, transmission, and movement execution (Figure 3). That is, the user perceives the car’s movements relative to the maze, plans a desired car trajectory, and then executes the appropriate wrist pitch and roll to maneuver the car efficiently. This process requires the user to quickly adapt to optimize the trade-off between car speed and accuracy (i.e., avoiding wall contacts) [30].

### 2.2. Human Subject Experiment

Eleven participants (healthy by self-report, male = 6, female = 5; right hand dominant = 10; 28.9 ± 5.6, 21–39 years old) with no prior GC-Rebot experience provided their informed consent to participate in this study. The study was conducted in accordance with the Declaration of Helsinki, and the protocol was approved by the Institution’s Ethical Review Committee. To categorize the participants’ spatial mapping skill level, they rated their video-game experience as either none (*n* = 1), novice (*n* = 5), or experienced (*n* = 5). These categories will be used throughout the paper exclusively to group participants based on their prior video-game experience.

**Figure 4:**
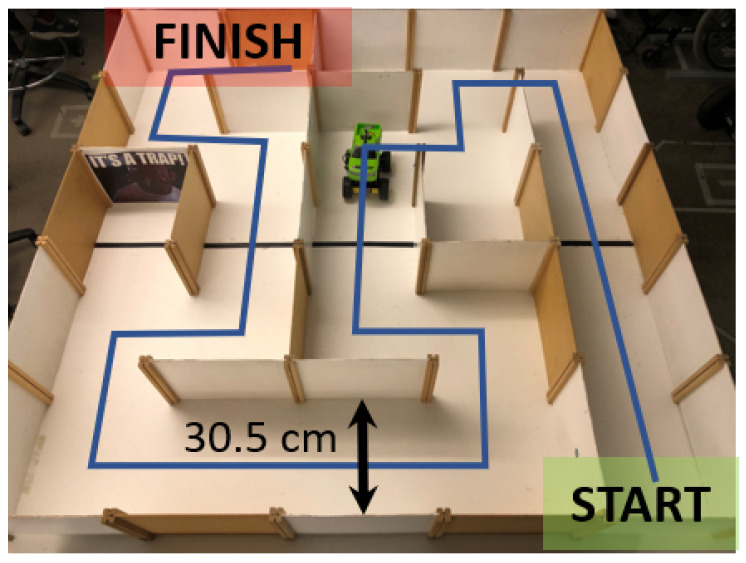
The maze executed by all participants was completed in the same direction (start to finish) for each trial. The blue line along the maze pathway (wall width = 30.5 cm; dowel protrusion = 0.75 cm each) depicts the nominal route, consisting of 14 straight segments and 13 total 90° turns (6 left, 7 right).

The gesture controller was secured to each participant’s hand using a fitted glove and elastic over wrap. This fitting involved first placing the Xsens IMU in the small pouch sewn to the dorsal side of the glove provided by the manufacturer. Then, the gesture controller was mounted in parallel and secured with the over wrap. After brief instruction on gesture-controller operation, participants were given a one-minute practice session outside of the maze. For the performance tests, they were instructed to navigate the test maze (Figure 4) as fast as possible, beginning each trial with their hand positioned flat relative to the floor. Participants were free to move around the maze as needed. Trials began with an audible command to start and were considered complete when the car made contact with the wall at the finish. Prior to conducting subsequent trials, the car was returned to the initial starting location. Participants performed three consecutive trials starting with either their dominant or non-dominant hand, which was randomized across participants. After completing the first three maze trials, the gesture controller was switched to the participant’s opposite hand; they then repeated the one-minute practice session outside of the maze, followed by three additional maze trials.

### 2.3. Questionnaires

All participants completed the validated System Usability Survey for technology-based applications [32], which consisted of 10 questions that asked participants to rate the system’s usability on a five-point Likert scale [33] ranging from strongly disagree (1) to strongly agree (5). The overall score (average across 10 questions) indicates perceived system usability. Participants also rated the level of effort used for each hand on a five-point Likert scale ranging from exhausting (1) to effortless (5). In addition, participants self-reported their dominant hand as their preferred writing hand.

### 2.4. Data Analysis

Xsens IMU pitch and roll angle and angular velocity signals were demeaned and used to quantify movement quality and quantity for each trial. Mean wrist active range of motion was defined as the average pitch or roll angular excursion (i.e., flexion-extension/pronation-supination) across each trial. Movement smoothness was quantified as the natural log of dimensionless hand angular acceleration (${\ddot{\theta }}_{lnD}$), which was adapted from [34], as follows:

(3) $${\ddot{\theta }}_{lnD}=-ln\left(\frac{{\left(t_{2}-t_{1}\right)}^{2}}{{\dot{\theta }}_{peak}^{2}}\int_{t_{1}}^{t_{2}}{\left|\ddot{\theta }\left(t\right)\right|}^{2}dt\right)$$

where ${\dot{\theta }}_{peak}$ is the maximum angular velocity and $\ddot{\theta }\left(t\right)$ is the first time-derivative of angular velocity. The hand angular accelerations were chosen as a proxy for smoothness because they map to car jerk, just as hand orientations map to car velocities. As improved motor coordination in patients post-stroke can be linked to reduced jerk [34,35], minimizing car jerk through reduced hand angular accelerations may imply improved movement performance. Total angular excursion ($\theta_{\mathrm{Tot}}$) was also quantified as the summed angular trajectory across each trial, where $\dot{\theta }\left(t\right)$ is the angular velocity,

(4) $$\theta_{Tot}=\int_{t_{1}}^{t_{2}}\left|\dot{\theta }\left(t\right)\right|dt$$

Movement quantity for each trial was quantified as the number of movement repetitions, dose-rate (^reps^/_min_), and total task duration. Movement repetitions were quantified by the peaks in the angular velocity signal, which was smoothed with a 2 Hz cutoff 4th-order Butterworth filter. Finally, the potential for engagement was assessed through system usability and perceived effort survey scores.

A two-way analysis of variance (ANOVA, α=0.05, p< 0.05) was used to detect mean differences in outcome metrics for two independent factors (trial number and video-game experience). Experienced (*n* = 5) and novice (*n* = 5) categories were compared in the analysis. A similar ANOVA was performed to test differences between effort score with two independent factors (hand dominance and video-game experience) and to test differences between usability survey scores with one independent factor (video-game experience). To demonstrate the learning effect, differences by video-game experience, trial duration, and either the number of repetitions, total excursion, smoothness, or dose-rate for the first and last trials are reported as 75% confidence interval ellipses. Correlation coefficients (*r*) [36] and coefficients of determination ($r^{2}$) [37] were also calculated across all trials to quantify the strength of these relationships. As only one participant reported no video-game experience, these results were assessed separately.

**Figure 5:**
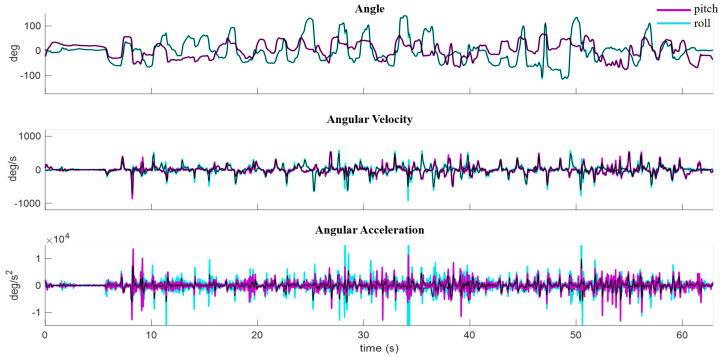
XSens IMU filtered (black) and unfiltered pitch (magenta) and roll (blue) angle, angular velocity, and angular acceleration signals versus time for a single trial.

A frequency response analysis was performed to assess future data collection rates required for the GC-Rebot. The Xsens IMU pitch and roll angle, angular velocity, and angular acceleration signals were low pass filtered (2nd-order Butterworth, 5 Hz cutoff) to estimate the effects of reducing the sampling rate to 10 Hz, which is equivalent to the GC-Rebot’s on-board IMU transmission speed to the car platform (Figure 5). A fast Fourier transform (FFT) was conducted to assess the frequency content of the IMU signals, and mean differences between the filtered and unfiltered metrics were quantified. The subsequent analysis was completed with the unfiltered signals.

## 3. Results

### 3.1. Quantitative Performance Metrics

**Figure 6:**
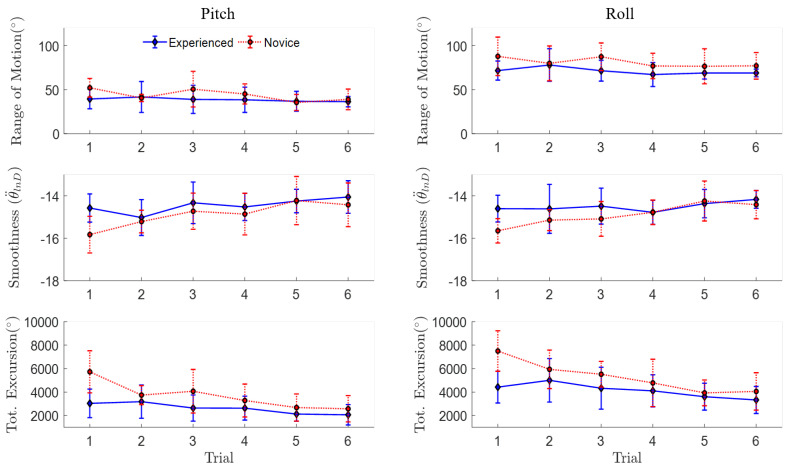
Mean ± SD quality of movement IMU-based metrics across six repeated trials including pitch and roll active range of motion (°), smoothness (${\ddot{\theta }}_{lnD}$), and total angular excursion (Tot. Excursion: $\theta_{Tot}$ (°)) separated by video-game experience (experienced, novice).

**Figure 7:**
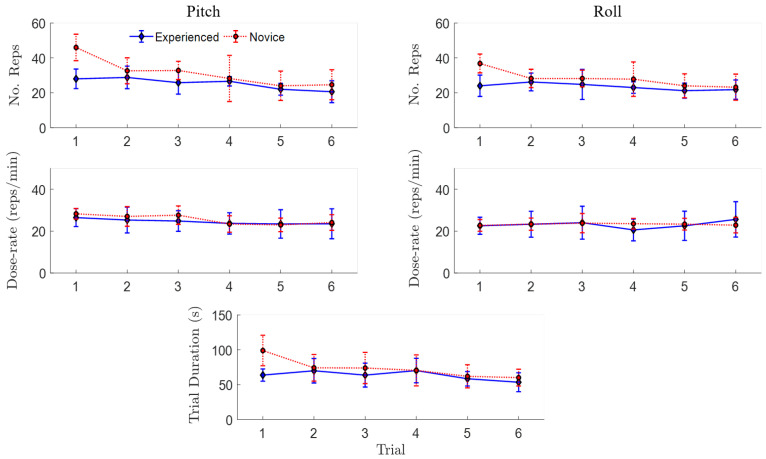
Mean ± SD quantity of movement IMU-based metrics across six repeated trials including pitch and roll number of repetitions (No. Reps), dose-rate (^reps^/_min_), and trial duration (s), separated by video-game experience (experienced, novice).

The mean and standard deviation (mean ± SD) movement quality metrics across participants with video-game experience (*n* = 10) revealed relatively consistent pitch and roll active range of motion across trials, with 35 degrees less overall pitch range of motion compared to roll (see the first row in Figure 6 and Table 2). Compared to the experienced participants, the novice group showed larger changes in both total angular excursion and pitch angular smoothness between the first and last trials (Figure 6, Table 2). A larger learning effect was also observed for movement quantity of the novice participants compared to the experienced group (Figure 7, Table 3). For example, novice participants performed fewer repetitions and had a shorter trial duration to complete the last trial compared to the first; however, dose-rates for pitch and roll remained consistent for both novice and experienced participants, with an overall average of 24.2 ± 5 ^reps^/_min_ across trial and movement (Table 3).

**Figure 8:**
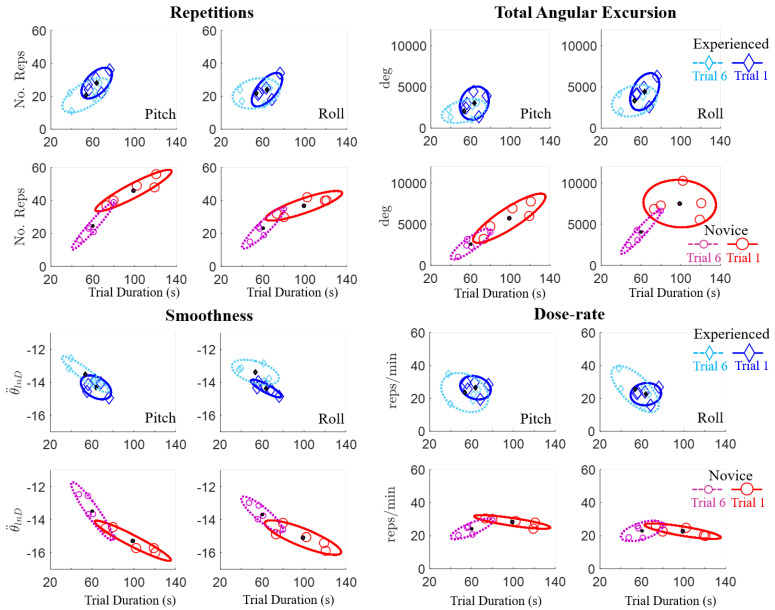
Motor learning differences of experienced and novice participant groups. Motor performance metrics including the number of repetitions (No. Reps), total angular excursion (Tot. Excursion: $\theta_{Tot}$ (°)), smoothness (${\ddot{\theta }}_{lnD}$), and dose-rate (^reps^/_min_) for pitch and roll motions are plotted versus trial duration (s) for the first (Trial 1) and last (Trial 6) trials. Individual participant performance (diamond and circle markers) and sample-distribution ellipses (75% confidence intervals) are also shown.

**Figure 9:**
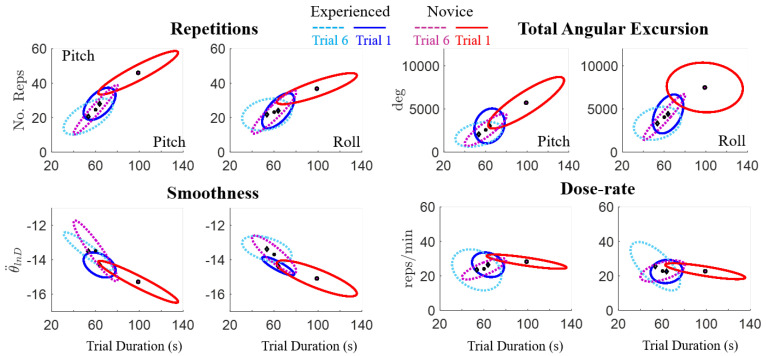
Sample-distribution ellipses (75% confidence intervals) for novice and experienced participants from Figure 8 are overlaid to demonstrate the convergence of the participant groups’ motor performance by Trial 6, based on the overlapping sample-distribution ellipses.

Focusing only on the first and last trials, the qualitative differences in motor learning between participants can be observed in Figure 8. The novice participants demonstrated larger decreases in trial duration, which were associated with larger reductions in the number of repetitions and total angular excursion, as well as larger increases in angular smoothness compared to the experienced group. Furthermore, by the last trial, the novice participants’ motor performance was similar to that of the experienced participants, as shown by the overlapping Trial 6 sample-distributions in Figure 9. Across all trials, the correlation coefficients ranged between 0.9 and 0.93 between trial duration and either the number of repetitions, total pitch angular excursion, or smoothness for the novice participants, which is significant (Table 4). These correlations corresponded to coefficients of determination ($r^{2}$) that imply 80–86% of the variance in movement quality/quantity can be explained by the variation in overall performance (time duration) [37]. However, the only potentially linear relationship for the experienced participants was between trial duration and smoothness. Dose-rate was weakly correlated with trial duration for both groups ($r^{2}$ < 0.35).

**Figure 10:**
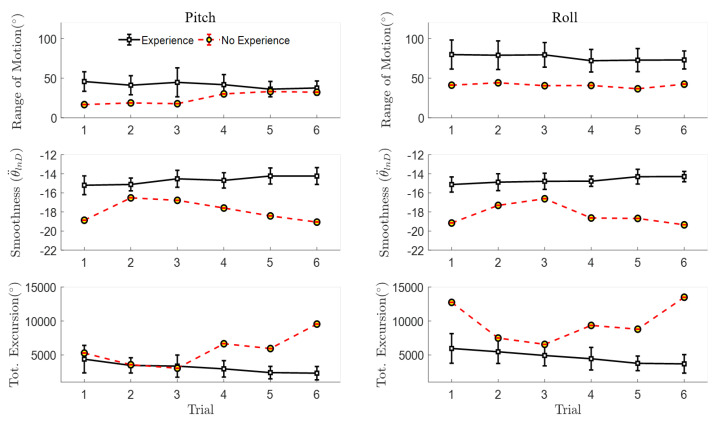
Quality of movement metrics across six repeated trials for a single participant with no video-game experience compared to the mean ± SD across the participants with video-game experience.

**Figure 11:**
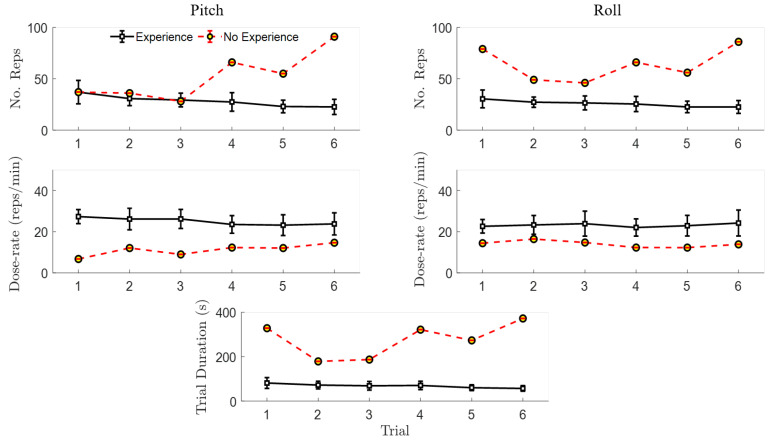
Quantity of movement metrics across six repeated trials for a single participant with no video-game experience compared to the mean ± SD across the participants with video-game experience.

The quality of motor performance of the participant with no prior video-game experience differed from the other participants (Figure 10). Across trials, this participant performed smaller active ranges of motion (pitch: 24.9 ± 8, roll: 40.9 ± 3), had less smooth angular movements (pitch and roll: −17.5 ± 1), and had greater total angular excursion (pitch: 5680 ± 2350°, roll: 9740 ± 2800°). This participant’s quantity of movement also differed from the other users (Figure 11), with consistently more repetitions (pitch: 52 ± 24 reps, roll: 64 ± 16 reps), longer trial duration (277 ± 79 s), and lower dose-rates (pitch: 11.1 ± 3 ^reps^/_min_, roll: 14.0 ± 2 ^reps^/_min_) across trials.

### 3.2. Signal Processing

**Figure 12:**
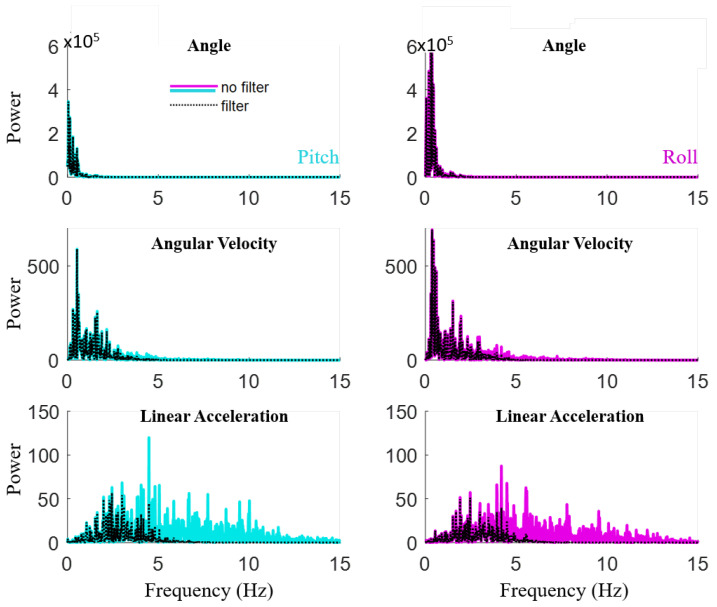
Fast Fourier transforms (FFT) comparing filtered and unfiltered Xsens IMU signals demonstrate that a slower sampling rate would likely be sufficient for the angle and angular velocity-based metrics because the frequency contents are below the low pass filter 5 Hz cutoff.

Most of the frequency content of the Xsens IMU pitch and roll angles and angular velocities were well below the 5 Hz low pass filter cutoff (Figure 12). Therefore, applying a 5 Hz filter to this dataset produced only small differences in the mean outcome metrics (0.2–5%) compared to the unfiltered data and no change in statistical correlations. Although larger effects were demonstrated for linear acceleration (Figure 12), this signal was not included in the assessments of movement performance.

### 3.3. Surveys

**Figure 13:**
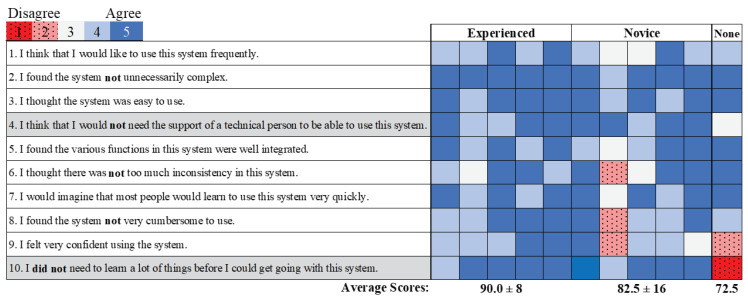
System usability survey scores separated by participant (*n* = 11). Average scores are separated by video-game experience (experienced, novice, none). The survey items highlighted in gray are specifically associated with learnability. The survey statements were converted to the positive direction (changes in bold font) to be consistent with scoring and facilitate the interpretation of the figure.

**Figure 14:**
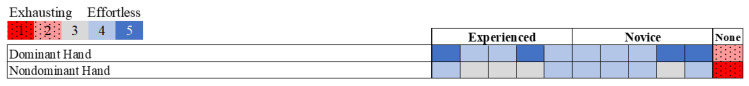
Effort scores separated by participant (*n* = 11), hand dominance, and video-game experience (experienced, novice, none). The participant without video-game experience found the task to be exhausting.

The average system usability scores across the participants with video-game experience (86.3 ± 12) corresponded to a rating of excellent [38]. Differences were not detected in the usability score between the novice and experience participants (*p* = 0.4); however, the novice scores were twice as variable (Figure 13). Separating the survey questions into use (eight items) and learnability (two items) categories [39,40], two of the three responses by the participant with no video-game experience were associated with learnability, contributing to the lower than average usability score (72.5) (Figure 13). This participant also reported that the effort to complete the task was exhausting (1) and near exhausting (2) for his/her non-dominant and dominant hands, respectively (Figure 14). These ratings corresponded to 62% and 41% more effort compared to the effort ratings reported by the experienced and novice participants. However, those with experience also reported greater effort with their non-dominant hand (2.6 ± 0.5) compared to their dominant hand (3.4 ± 0.5), which is significant (*p* = 0.004).

## 4. Discussion

The GC-Rebot system, which uses coordinated hand gestures to wirelessly control a motorized car through a maze, was assessed as a potential alternative to physical therapy. This study characterized movement performance through quantitative assessments of movement quality and quantity, which revealed a high dose-rate compared to standard physical therapy with mean active ranges of motion that were 30–50% of the typically available range (120°–160°). This intense execution of simple wrist movements replicates functional training, which is a key element to rehabilitation that forms a foundation for normal movements [7]. Differences in motor learning and system learnability between participants with varying levels of hand-eye coordination experience suggest that user-specific challenge levels could promote learning and satisfaction, which could be leveraged for treatment. Specifically, altered maze courses, tunable controller gains, and adjusted deadbands could elicit various challenges and ranges of motion dependent on the user’s therapy goals. In addition, this versatile platform affords attachment to different body segments to expand therapy to other joints (e.g., ankle, elbow) and has the potential to accommodate participant-specific neutral positions and gesture thresholds for varied limb impairments.

### 4.1. Quantified Performance

The movement performance metrics produced objective assessments that characterized user behavior while conducting GC-Rebot training. The average participant performed 180 full wrist motion repetitions over a 5–10 min session (six trials with approximately 30 repetitions each). The corresponding average dose-rate (24 ^reps^/_min_) represents an almost seven-fold increase in the dose-rate achieved during active exercise repetitions in a standard 30 min therapy session (0.5–3.5 ^reps^/_min_) [4]. These results suggest that a 25-min training session with GC-Rebot could attain the adequate dosage (300–400 repetitions) to promote neuroplastic motor recovery [1]. Furthermore, this session duration is half the time compared to a study of stroke survivors who achieved 322 reps in a 60-min therapy session [41]. In Birkenmeier et al. [41], therapists tracked the number of repetitions and rated task performance to identify when to increase the level of challenge. Sensor-based technology such as GC-Rebot can automate measurements of movement performance, impairment, and recovery progress outside the clinic to ease the burden on therapists and increase assessment frequency, sensitivity, and resolution [34].

### 4.2. Skill-Based Motor Learning

Beyond a high dosage through repetitive movement, brain reorganization also requires learning [1]. Our study results demonstrate that GC-Rebot training of a novel wrist coordination task elicits motor learning, with increased rates for novice users. For example, faster maze completion times were correlated with increased movement smoothness and reduced total angular excursion. Grouping the data across user and trials contributed to the relatively high variation in these quantitative metrics (25% of the mean on average across metrics), which is similar to the variation in wrist movements related to a goal-directed, voluntary task in a virtual reality environment [42]. This varied user performance likely relates to skill level and strategy differences and is consistent with a prior study, which suggested between-participant performance is highly variable [43]. Furthermore, wrist rotation coordination demonstrates more variability compared to the more commonly targeted gross proximal movements associated with reaching tasks [44]. Minimizing user performance variation with an optimal level of challenge could leverage motor learning and enhance engagement.

A game therapy approach promotes implicit learning [45] based on the intrinsic feedback to the user through self-evaluation on task performance and enhanced motivation [1,46]. Furthermore, this high-frequency, concurrent feedback on a relatively complex task has been suggested to be effective for learning, potentially through the automation of movement control [46,47]. Applying these techniques to wrist therapy, which is a less frequently targeted treatment by robotic rehabilitation systems, has the potential to reduce impairment because functional gains in upper extremity movements are dependent on coordinated distal motion (i.e., wrist/hand) [45]. For example, a robotic system that targeted wrist motion reduced motor deficits, quantified by increased wrist extension and improved functional survey scores [42]. Furthermore, the relative novelty of a gesture-controlled training paradigm could promote initial interest by minimizing preconceived expectations of successful performance. In contrast to performing a task that was easily mastered prior to the impairment, this approach may avoid initial frustration and promote engagement. As a supplement to the standard of care and targeted task-specific training, the potential benefits of GC-Rebot therapy lie in the targeting of coordinated wrist movements and implicit learning of the underlying capabilities required for many functional tasks; however, the direct translation to improved function, particularly within an impaired population, remains an area for future study.

The participant with no video-game experience was less skilled and further challenged by GC-Rebot training compared to the other users, performing the task with 43% less active range of motion and 19% less smooth movements (i.e., more negative), producing a four-fold increase in trial times and 45% and 60% more pitch and roll repetitions, respectively. The dose-rate was also 48% smaller across trials for this participant. The consistently reduced performance from this less-skilled individual corresponds to 51% greater perceived effort across limbs and a 16% lower usability score, which may negatively affect compliance and motor learning in a physical therapy application. These results suggest that dose-rate, movement quality, perceived effort, and engagement are related to skill level and that tailoring the task to individual ability could optimize training effects. That is, these quantitative movement metrics collected during the trials have great potential to guide user-specific settings for achieving adequate and progressive levels of challenge to leverage motor learning [28]. For example, matching the user’s skill level to task difficulty can prevent frustration, boredom, and fatigue [5], which is important for promoting motor learning and engagement [10], especially for users with physical and cognitive impairments [48]. However, further study of the relationship between prior experience and subsequent motor learning with a larger sample population are required to confirm these findings. Finally, as treatment progresses, resistance to wrist motion or grasping real-world household objects (e.g., pencil, hammer) could be added to further address strength and dexterity deficits. Associating movement performance metrics with the appropriate cognitive and physical challenge levels during a therapy session and throughout the course of treatment for maximized motor learning is an important extension of this work.

### 4.3. Engagement: Usability and Effort

An affordable, easy-to-use, and entertaining form of physical therapy that promotes motor learning and can be conducted in an at-home environment is critical for prolonged and effective treatments. These programs are especially important in the long-term care of people post-stroke, where arm-hand recovery post-stroke lags other functions [49]. With a total cost of less than $200 and “excellent” user ratings according to [38], GC-Rebot demonstrates potential as an engaging, intuitive telerehabilitation solution to address the cost-prohibitive nature of prolonged therapy [4]. The usability survey was chosen because it is reliable with small samples (8–12 people) and has become the industry standard [38]. However, some inherent bias in participant responses may exist due to the unblinded study design. One way to improve usability is to minimize the number of sensors donned by the user by eliminating the requirement of a separate IMU. This system simplification can be realized by adding data acquisition to the car platform for on-board IMU data analysis. The FFT analysis confirms that the 10 Hz wireless transmission between the gesture controller and car is sufficient to quantify performance and motor learning while conducting GC-Rebot training.

The positive user experience was associated with a task that required effort, especially when performed with the non-dominant hand. This effort corresponded to the repetitive active range of motion and neuromuscular coordination required to control the car, which can be adapted according to an individual’s impairment level. For example, the present maze produced pitch excursions that were on average 30% of the typically available range of motion (120°–160°), due to its short, forward, straight segments. However, larger roll excursions (50% of the typically available range of motion) were required for turns. Longer, straight segments with backward driving requirements and adjusted proportional control with a deadband could increase the user’s active range of motion. Alternatively, fewer turns and reduced controller thresholds could lessen the challenge, which may be appropriate to promote engagement and motor learning for users with less experience [28] or neuromusculoskeletal deficits [5].

Future work should examine the system’s ability to promote therapy compliance toward improved motor performance in an impaired population. For example, gesture-controlled game therapy may alleviate precision and coordination deficits in people post-stroke through targeted visuospatial coordination and motor planning rehabilitation [50]. Inspired by the potential for improved functional outcomes with video-game (2D) or virtual reality-based (non-physical environment) therapy [5,15,16], the GC-Rebot involves a 3D, physical environment with spatial mapping concurrent feedback, which may alter the user’s perceptual input, planning, and associated motor control [51]. For example, learning tai chi movements with a 3D immersive system was more effective compared to a 2D video [52]. These findings suggest that a task performed in a 3D environment can elicit different motor learning and functional outcomes; therefore, further research is warranted to confirm whether motor learning and improved function can be achieved through GC-Rebot therapy.

## 5. Conclusions

Through intuitive gesture control, the GC-Rebot system provided quantitative assessments of movement performance with a user-friendly and engaging activity, which may promote therapy compliance. Enhanced engagement, affordability, and a high dose-rate support the GC-Rebot’s potential as an effective tool for evidence-based at-home rehabilitation.

## Figures and Tables

**Table 1:** GC-Rebot gesture controller and motorized car specifications

Part Type	Description	Details
	**Gesture Controller**	Total Cost: $60
IMU	Adafruit BNO055 9-DOF	5 V, 16 MHz
MCU	Adafruit ItsyBitsy (Atmega32u4)	5 V, 16 MHz
Transceiver	NRF24L01 wireless module	1.9–3.6 V, 2.4 GHz
Battery	Adafruit lithium ion polymer	3.7 V, 150 mA
	**Motorized Car**	Total cost: $75
Car Platform	SZDOIT Smart Robot Car Kit	2 mm alum.panel
Motors	4 TT DC Gearbox Motors (1:48)	4.5 V, 200 RPM
Motor Driver	Quad DC motor driver shield	SKU:DRI0039
MCU	Arduino Uno R3 (ATmega328P)	5 V, 16 MHz
Transceiver	NRF24L01 wireless module	1.9–3.6 V, 2.4 GHz

**Table 2:** Movement quality performance and learning differences for participants with video-game experience (*n* = 10). Descriptive statistics (mean ± SD) are presented across participants and trials. Motor learning was compared between the novice (ΔNov, *n* = 5) and experienced (ΔExp, *n* = 5) groups by evaluating each group’s change in metric between the first and last trials. The corresponding percent differences (%Diff) are also included. Significant differences (*p* < 0.05) are indicated with bold font.

Metric		Mean ± SD	ΔNov	%Diff	ΔExp	%Diff	*p*-Value
Active Range of Motion (°)	Pitch	41.6 ± 13	−11.8	−28%	−1.3	−3.1%	0.3
	Roll	76.8 ± 16	−12.9	−17%	−3.4	−4.4%	0.2
Smoothness (${\ddot{\theta }}_{lnD}$)	Pitch	−14.1 ± 0.9	1.8	13%	0.80	5.7%	**0.04**
	Roll	−14.1 ± 0.7	1.4	10%	0.99	7.0%	0.4
Total Ang. Excursion: $\theta_{Tot}$ (°)	Pitch	3150 ± 1490	−3170	−101%	−967	−31%	**0.03**
	Roll	4710 ± 1770	−3440	−73%	−1090	−23%	**0.01**

**Table 3:** Movement quantity performance metrics and learning differences for participants with video-game experience (*n* = 10). Descriptive statistics (mean ± SD) are presented across participants and trials. Motor learning was compared between the novice (ΔNov, *n* = 5) and experienced (ΔExp, *n* = 5) groups by evaluating the change in metric between the first and last trials. The corresponding percent differences (%Diff) are also included. Significant differences (*p* < 0.05) are indicated with bold font.

Metric		Mean ± SD	ΔNov	%Diff	ΔExp	%Diff	*p*-Value
Number of Repetitions	Pitch	28.5 ± 9	−20.8	−73%	−7.4	−26%	**0.04**
	Roll	25.8 ± 7	−13.4	−52%	−2.0	−7.8%	**0.02**
Dose-rate (^reps^/_min_)	Pitch	25.1 ± 5	−3.8	−15%	−2.7	−11%	0.9
	Roll	23.2 ± 5	0.25	1.1%	3.2	14%	0.4
Trial duration (s)		68.3 ± 19	−38.9	−57%	−10.3	−15%	**0.03**

**Table 4:** Correlation coefficients (*r*) across all trials quantifying the strength of the relationship between trial duration (s) and motor performance metrics for novice and experienced participants. Significant differences (*p* < 0.05) are indicated with bold font.

Metric		Novice		Experienced	
		*r*	*p*-**Value**	*r*	*p*-**Value**
Number of Repetitions	Pitch	0.909	**0.0000**	0.613	**0.0003**
	Roll	0.906	**0.0000**	0.363	0.05
Total Ang. Excursion (°)	Pitch	0.910	**0.0000**	0.407	**0.03**
	Roll	0.685	**0.0000**	0.383	**0.04**
Smoothness (${\ddot{\theta }}_{lnD}$)	Pitch	−0.929	**0.0000**	−0.771	**0.0000**
	Roll	−0.896	**0.0000**	−0.831	**0.0000**
Dose-rate (^reps^/_min_)	Pitch	0.102	0.6	−0.430	**0.02**
	Roll	−0.356	0.05	−0.582	**0.0008**
